# Autologous platelet concentrate for the treatment of macular hole: a systematic review and meta-analysis

**DOI:** 10.31744/einstein_journal/2024RW0832

**Published:** 2024-11-12

**Authors:** Dillan Cunha Amaral, Mário Luiz Ribeiro Monteiro, Milton Ruiz Alves, Ivar Vargas Belizario, Lucas de Sousa Tebicherane, Raíza Jacometti, José Eduardo Ferreira Manso, Agma Juci Machado Traina, Ricardo Noguera Louzada

**Affiliations:** 1 Universidade Federal do Rio de Janeiro Faculdade de Medicina Rio de Janeiro RJ Brazil Faculdade de Medicina, Universidade Federal do Rio de Janeiro, Rio de Janeiro, RJ, Brazil.; 2 Universidade de São Paulo Faculdade de Medicina Divisão de Oftalmologia e Laboratório de Investigação em Oftalmologia São Paulo SP Brazil Divisão de Oftalmologia e Laboratório de Investigação em Oftalmologia (LIM-33), Faculdade de Medicina, Universidade de São Paulo, São Paulo, SP, Brazil.; 3 Universidade de São Paulo Faculdade de Medicina Ribeirão Preto SP Brazil Faculdade de Medicina, Universidade de São Paulo, Ribeirão Preto, SP, Brazil.; 4 Universidade de São Paulo Instituto de Ciências Matemáticas e de Computação São Carlos SP Brazil Instituto de Ciências Matemáticas e de Computação, Universidade de São Paulo, São Carlos, SP, Brazil.

**Keywords:** Macular hole, Autologous platelet, Vitrectomy, Retinal perforations

## Abstract

Autologous platelet concentrates are rich in growth factors and have shown potential for high anatomical success rates in macular hole treatment. However, no systematic review has yet assessed its impact. Therefore, this study aimed to conduct a comprehensive review and meta-analysis on the comparative efficacy and safety of autologous platelet concentrates in treating macular hole. A systematic review of the literature on autologous platelet concentrate therapy for macular hole was conducted in accordance with Preferred Reporting Items for Systematic Reviews and Meta-Analysis (PRISMA) guidelines on September 9, 2023. Databases, including PubMed, the Cochrane Library, Web of Science, and Embase, were queried. A meta-analysis of random effects was performed. Efficacy was evaluated through anatomical closure, reopening of the macular hole at 6 months, average improvement of 2 lines, and visual field loss. Safety was evaluated by monitoring complications. A systematic search of multiple databases identified six studies (three randomized controlled trials and three non-randomized cohort studies) involving 616 patients (626 eyes). Autologous platelets concentrate therapy significantly improved macular hole closure compared to that in the controls (OR=4.35; 95%CI=2.08-9.10; p<0.0001; I²=9%). No significant differences were observed in hole reopening at 6 months, post-operative visual acuity improvement by 2 lines or more, or visual field loss between autologous platelets concentrate and control groups. The overall complication rates were similar between groups. Thus, autologous platelet concentrate therapy shows promise for promoting macular hole closure, particularly in smaller holes. Further research with standardized protocols, prolonged follow-ups, and larger sample sizes is needed to fully understand its benefits and limitations in macular hole closure.

**Prospero database registration:**

(https://www.crd.york.ac.uk/prospero/) under ID CRD42023455815.

## INTRODUCTION

The first description of a macular hole (MH) emerged in 1869, which attributed its genesis to a traumatic incident.^(1,2)^ MHs are characterized by a vertical defect in the neurosensory retinal anatomy, primarily within the foveal region, extending from the internal limiting membrane (ILM) to the retinal pigment epithelium (RPE).^(3–6)^ It profoundly affects central vision and induces metamorphopsia. Most MHs are primary and idiopathic, whereas secondary MHs can be linked to factors such as high myopia, trauma, proliferative diabetic retinopathy, and various retinal pathologies.^(7)^ The gold standard for treating MH is surgical intervention involving pars-plana vitrectomy (PPV), induction of posterior vitreous detachment, and optionally, peeling of the internal limiting membrane (ILM) followed by gas tamponade.^(8–10)^ However, several techniques have been introduced to enhance surgical outcomes, including the inverted ILM flap,^(11,12)^ lens capsular flap transplantation,^(13)^ tapping of MH edges,^(14)^ autologous serum injection,^(15)^ human amniotic membrane, and autologous retinal transplantation.

Challenging cases, including large MHs, myopic MHs, and MHs associated with retinal detachment, are associated with poorer visual outcomes and lower closure rates.^(2)^ This has led to modifications in conventional MH surgery, such as the use of autologous platelet concentrate (APC) for enhanced success. Platelet extracts, which are rich in growth factors, have been proven effective in MH treatment, achieving high anatomical success rates.^(16–19)^ Despite the potential benefits of APC, its impact on MH treatment has not been systematically reviewed.

## OBJECTIVE

This study aimed to assess the comparative efficacy and safety of autologous platelet concentrate for treating macular hole.

## METHODS

### Eligibility criteria

Inclusion in this meta-analysis was restricted to studies that met the following eligibility criteria: human study; participants: people with MH; comparison of APC therapy with control; at least one or more clinical outcomes representing intraoperative and/or postoperative outcome parameters assessed and published; design: randomized trials or nonrandomized cohorts; and reports of any clinical outcomes of interest. We excluded studies with no control group, studies on animal or cadaver subjects, and reports not published in English.

### Search strategy and data extraction

The protocol for this systematic review of literature on APC therapy for MH surgery is registered in the PROSPERO International Prospective Register of Systematic Reviews, following the Preferred Reporting Items for Systematic Reviews and Meta-Analysis (PRISMA) guidelines for protocol data extraction. The term ("Retinal Perforation" OR "Holes, Retinal" OR "Macular Hole" OR "Macular Holes" OR "Retinal Break" OR "Retinal Breaks" OR "Retinal Dialyse" OR "Retinal Dialyses" OR "Retinal Hole" OR "Retinal Holes" OR "Retinal Perforation" OR "Retinal Tear" OR "Retinal Tears") AND ("Blood Platelet" OR Platelet OR "Platelet, Blood" OR Platelets OR "Platelets, Blood" OR Thrombocyte OR Thrombocytes) was used for the search. The search terms were queried in the PubMed (MedLine), Embase (Elsevier), Cochrane, and Web of Science databases. The searches started on July 20, 2023, and ended on August 20, 2023. References from all included studies, previous systematic reviews, and meta-analyses were also manually searched for additional studies. Two authors independently extracted the data using predefined search criteria and quality assessments.

### Statistical analysis

This systematic review and meta-analysis were performed in accordance with the Cochrane Collaboration and PRISMA statement guidelines.^(20)^ Odds-ratios (OR) with 95% confidence intervals (95%CI) were used to compare the treatment effects for categorical endpoints. Continuous outcomes were compared using standardized mean differences. Heterogeneity across studies was evaluated using Cochran's Q test, I^2^ test, and τ^2^ test. An I^2^ value greater than 50% was considered indicative of high statistical heterogeneity, for which a random-effects model was used. A random-effects model was used for all analyses because of heterogeneity. Review Manager 5.3 (Cochrane Centre, The Cochrane Collaboration, Denmark) was used for the statistical analysis.

## RESULTS

### Study selection and characteristics

**Figure 1:**
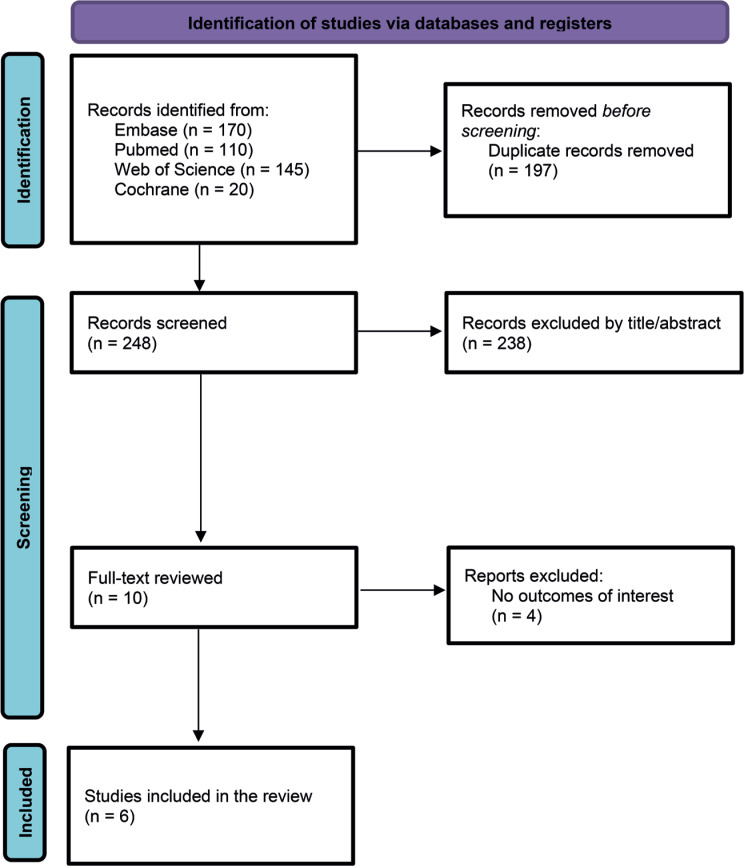
Study screening and selection process

As detailed in figure 1, 301 articles were found, with 110 from PubMed (Medline), 170 from Embase (Elsevier), 145 from Web of Science, and 20 from the Cochrane databases. Among these, 197 were excluded as duplicates. After removing duplicate records and ineligible studies, 10 studies remained and were fully reviewed based on the inclusion criteria. Four articles were excluded based on the exclusion criteria. Finally, six studies were included in the review: three randomized controlled trials (RCTs)^(17,21,22)^ and three non-randomized cohort studies.^(16,23,24)^

In total, 616 patients (626 eyes) were included in this study. The mean age was 65.01±3.45 years in the platelet arm and 65.51±1.55 in the control arm. There were 273 eyes in the platelet arm and 353 in the control arm. The minimum aperture size measurement of the MH was traced in all studies at the level of the narrowest neurosensory retinal aperture between the MH edges, roughly parallel to the RPE/Bruch membrane complex.^(3)^ In the postoperative period, patients were required to stay as far as possible in the prone position (face-down) for 3-14 days.^(25)^ Retinal detachment, pigment changes in the macula and epiretinal membrane, keratoconjunctivitis, cataracts, elevated intraocular pressure, and endophthalmitis were the complications reported in the selected studies. The study characteristics are reported in table 1, and the pooled analysis of all studies is reported in table 2.

**Table 1:** Baseline characteristics of included studies

Author (year)	Study type	Participants [eyes]	Country	Mean age	Gender (F/M)	Base diameter of the hole (*μ*M) [platelet]	Base diameter of the hole (*μ*m) [control]	Follow-up
Gaudric et al (1995)^(16)^	OB prospective	40 [40]	France	65.90	NA	NA	NA	6 months
Paques et al (1999)^(17)^	RCT	110 [110]	France	67.48	73/37	NA	NA	6 months
Kim et al (2021)^(21)^	RCT	117 [117]	South Korea	64.19	34/83	1078.3±49.9	967.0±42.7	6 months
Babu et al (2020)^(22)^	RCT	60 [60]	India	61.85	40/20	1395.17±240.57	1486.90±281.61	3 months
Minihan et al (1997)^(23)^	OB prospective	75 [85]	Ireland	65.00	NA	NA	NA	6 months
Shpak et al (2021)^(24)^	OB retrospective	214 [214]	Russia	66.58	183/31	883±386	817±272	12 months

OB: observational; RCT: randomized control trial; F: female; M: male; NA: not applicable.

**Table 2:** Summary pooled analysis of all outcomes

Outcome	OR; 95%CI	p value	Heterogeneity I^2^ (%)
Anatomical closure	4.35; 2.08-9.10	<0.0001	9
Reopening in 6 months	1.84; 0.16-20.98	0.623366	NA
Average improvement of two lines or more	1.30; −0.15-0.24	0.68	68
Visual field loss	2.27; 0.79-6.57	0.13	NA
Complications	0.57; 0.17-1.97	0.375	81

P values less than 0.05 were considered statistically significant. OR: odds ratio; 95%CI: 95% confidence interval; I^2^: an I^2^ value greater than 50% was considered indicative of high statistical heterogeneity (NA% indicates that only one study had a positive outcome in those cases); NA: not applicable.

### Pooled analysis of all studies

**Figure 2:**
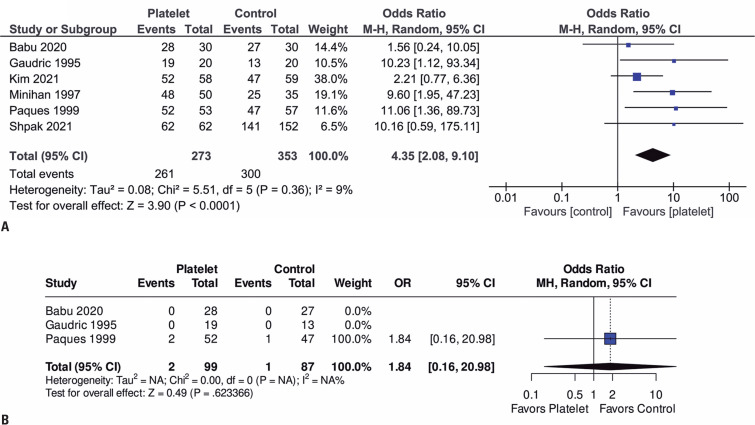
Forest plots of (A) anatomical closure and (B) reopening of the MH in 6 months

Compared with the control group, those receiving APC showed better results towards anatomical closure of the MH (OR=4.35; 95%CI=2.08-9.10; p<0.0001; I²=9%; Figure 2A). However, among those receiving APC, no significant difference was found compared with the control group in reopening of the MH in 6 months (OR=1.84; 95%CI=0.16-20.98; p=0.623366; I²=NA%; Figure 2B).

**Figure 3:**
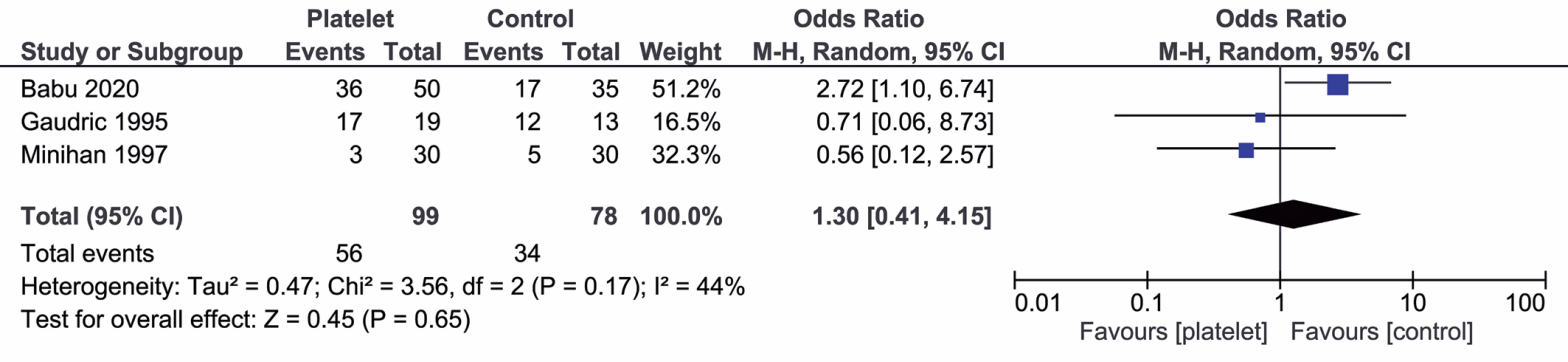
Forest plot of average improvement of 2 lines or more

No statistically significant difference was found between groups in the post-operative visual acuity indicated by an average improvement of 2 lines or more (OR=1.30; 95%CI=-0.15-0.24; p=0.68; I²=68%; Figure 3).

Furthermore, 16 visual field loss cases were reported in two studies,^(17,23)^ however, the occurrence of visual field loss was not significantly different between the platelet and control groups (OR=2.27; 95%CI=0.79-6.57; p=0.13; I²=NA%).

**Figure 4:**
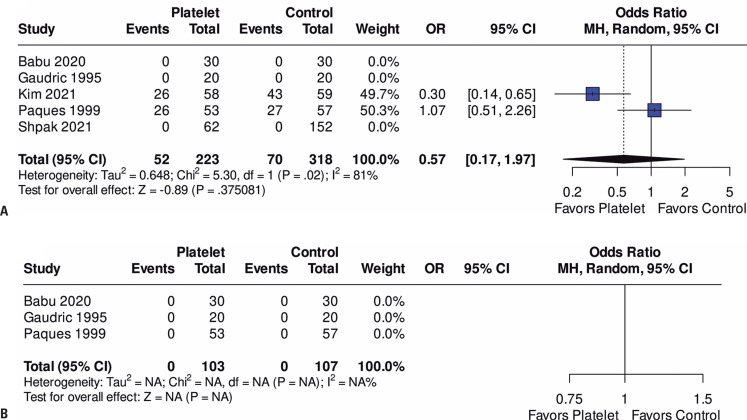
Forest plots of (A) Complications and (B) Endophthalmitis

Across the pooled studies, a total of 122 complications were observed among the 616 patients (626 eyes). The rates of complication (OR=0.57; 95%CI=0.41-4.15; p=0.65; I²=44%; Figure 4A) were not significantly different between the groups. Composite endophthalmitis was not observed in these studies (Figure 4B).

## DISCUSSION

Vitrectomy with internal limiting membrane (ILM) peeling and gas or silicone oil tamponade is considered the gold standard for treating MHs. However, this method has several limitations.^(26)^ Complications such as intraocular infection, retinal displacement, progressive cataract, and retinal vascular occlusion are prevalent, especially in patients with preexisting risk factors such as diabetes or advanced age.^(27)^

In the realm of MH surgery, the choice of an optimal tamponade agent is of paramount importance, factoring in various considerations such as patient positioning, duration of compromised vision, air travel feasibility, and potential complications. These elements are intricately modulated by hole-specific variables such as size and chronicity. This systematic review explores the existing body of evidence, specifically focusing on the use of APC as a tamponade agent. By dissecting and analyzing this approach, a more comprehensive understanding of its potential benefits and drawbacks emerges, enriching the discourse surrounding tamponade options in MH surgery.

Upon examining baseline characteristics, a notable similarity emerged in the mean ages of the two groups. Notably, most patients in both the groups were 60 years or older at the beginning of the research. This observation underscores the comparability of groups in terms of age distribution, which is pivotal for ensuring a balanced assessment of the impact of APC treatment on MH closure rates.

The primary measure of surgical success was closure of the MH. Notably, the analysis revealed a more favorable outcome for the group treated with APC than for the control group, as indicated by the OR. Additionally, it is pertinent to highlight that this analysis demonstrated a low level of heterogeneity, with an I^2^=9%, indicating a notable consistency among the included studies. These findings underscore the potential efficacy of APC in promoting MH closure, and highlight the robustness and coherence of the collected evidence.

Furthermore, the outcome measures of visual acuity improvement by two lines or more and visual field loss also demonstrated a lack of disparity between the groups. This suggests that although APC treatment may positively influence hole closure, it may not necessarily translate into significant short-term visual acuity gains, as with other MH therapies.^(28)^

Nevertheless, the incidence of MH reopening after 6 months did not show a significant difference. This emphasizes the complexity of the factors influencing the long-term stability of hole closure, and suggests the need for continued investigation into optimizing treatment durability.^(29–31)^ The results obtained in the reopening of the MH differed from those of other studies. Schaub^(32)^ reported better outcomes in the APC group. It is important to note that the study population included patients with persistent MHs, which can lead to different results. Therefore, further investigation of these aspects is important.

When considering complications, our findings demonstrated that the overall complication rates were not significantly different between the two groups, which is reassuring as it suggests that the use of APC does not inherently introduce a greater risk of complications, as observed in other studies.^(33)^

Macular hole stage plays a significant role in the patient's prognosis, including postoperative closure of the MH and visual acuity. As shown in table 1, the smaller the MH, the better is the outcome. This is anticipated as MHs progress from stage 1 to stage 4, with a corresponding decline in visual acuity. Consequently, the rate of visual loss increases as the hole enlarges, particularly in stages 3 and 4 MHs. The findings indicate that stage 2 MHs have a much better prognosis than those at stages 3 and 4, which has been reported in other studies.^(34)^

Furthermore, APC production is resource-intensive and complex and requires blood centrifugation and separation. In addition, a highly aseptic environment is necessary because of the risk of infection during long-term preservation.^(35,36)^

Nevertheless, it is essential to highlight that satisfactory outcomes can still be achieved without APC. Therefore, ophthalmologists may not prioritize APC use as the primary surgical approach.^(2,37)^ Although APC may not be the universally preferred method, its use is recommended in cases with specific indications, such as giant MH, recurrent MH, persistent MH, MH associated with high myopia, MH associated with trauma, or MH associated with vitreomacular traction.

Medical classification systems help comprehend disease progression, predicting possible outcomes, and evaluating prognoses.^(38)^ The Gass classification is >30 years old but is still useful in informing patients about their prognosis through observation alone. Currently, research efforts are focused on identifying additional biomarkers correlated with the established standard classification based on measuring the minimum aperture size at the level of the neurosensory retina, classified as small (≤250*μ*m), medium (>25 to ≤400*μ*m), and large (>400*μ*m) MH and linked with postoperative outcomes.^(3,5,39,40)^ This evolution contributes to a more precise understanding of the disease and plays a pivotal role in supporting informed decision-making to enhance the overall quality of life for patients. This approach maximizes the benefits offered by the APC in these specific scenarios.

### Limitation of this meta-analysis

The duration of symptoms is a difficult parameter to evaluate precisely, and therefore, as reported in other studies^(41,42)^ it is directly related to progression of the MH diameter and worse prognosis. Another chronological factor is the postoperative evaluation of visual accuracy. Some studies have only utilized the 6 months mark post-surgery for evaluation. Others evaluated two, three, and six months and even one or two years after surgery. This information could help us properly understand the long-term effects of treatment.

In certain studies, within the analysis, missing standard deviations for visual acuity measurements and the use of diverse measurement scales introduced uncertainty and complexity. The absence of standard deviations impacts the result assessment, whereas varying scales hinder direct result comparison. This methodological variation can compromise data aggregation, interpretation, and practical applications. Recognizing these limitations is crucial when evaluating findings from studies using different measurement approaches.

The use of different methods of extraction for APC or even the process of applying it in the eyes of the patient can affect the final results.

## CONCLUSION

In summary, our meta-analysis provides valuable insights into the potential efficacy and safety of autologous platelet concentrate therapy as a promising intervention for addressing macular holes. The analysis reveals that autologous platelet concentrate therapy provides a significant advantage in promoting the anatomical closure of macular holes when compared to control interventions. While the results regarding macular hole reopening and post-operative visual acuity improvements showed no statistically significant differences between the autologous platelet concentrate group and the controls, the observed trend towards enhanced closure underscores the therapeutic potential of autologous platelet concentrate. Notably, the macular hole stage emerged as a critical factor influencing treatment outcomes, with smaller holes exhibiting better prognoses. Acknowledging the limitations inherent in the included studies, such as varying methodologies for autologous platelet concentrate preparation and administration, symptom duration, and follow-up duration, it becomes clear that further research, ideally with standardized protocols, prolonged follow-up, and larger sample sizes, is required to establish the full spectrum of benefits and limitations of autologous platelet concentrate in macular hole closure. In the pursuit of refining treatment approaches for macular holes, our meta-analysis contributes to the growing body of evidence supporting the role of autologous platelet concentrate therapy in enhancing anatomical closure, offering a stepping-stone for future investigations in this dynamic field.
